# Exosomes with low miR-34c-3p expression promote invasion and migration of non-small cell lung cancer by upregulating integrin α2β1

**DOI:** 10.1038/s41392-020-0133-y

**Published:** 2020-04-22

**Authors:** Wenjing Huang, Yanyan Yan, Yun Liu, Minting Lin, Jinxiang Ma, Wei Zhang, Jianwei Dai, Jiajun Li, Qiaoru Guo, Hubiao Chen, Bolat Makabel, Hong Liu, Chaoyue Su, Hong Bi, Jianye Zhang

**Affiliations:** 1 Guangdong Provincial Key Laboratory of Molecular Target & Clinical Pharmacology, School of Pharmaceutical Sciences and the Fifth Affiliated Hospital, Guangzhou Medical University, 511436 Guangzhou, Guangdong P.R. China; 2 Institute of Respiratory and Occupational Diseases, Collaborative Innovation Center for Cancer, Medical College, Shanxi Datong University, 037009 Datong, P.R. China; 3 School of Chinese Medicine, Hong Kong Baptist University, Hong Kong, P.R. China; 4 College of Public Health, Guangzhou Medical University, 511436 Guangzhou, Guangdong P.R. China; 5 Cancer Center of Datong, the Second People’s Hospital of Datong, 037005 Shanxi, P.R. China; 6 GZMU-GIBH School of Life Sciences, Guangzhou Medical University, 511436 Guangzhou, Guangdong P.R. China; 7 Xinjiang Institute of Materia Medica, 830004 Urumqi, P.R. China; 8 Department of Pathology, Shanxi Provincial People’s Hospital, 030012 Taiyuan, P.R. China; 9 Key Laboratory of Tropical Translational Medicine of Ministry of Education, Hainan Medical University, 571199 Haikou, P.R. China

**Keywords:** Tumour biomarkers, Lung cancer

## Abstract

Exosomes play critical roles in regulating various physiological and pathological processes, including immune stimulation, immune suppression, cardiovascular diseases, and cancers. Recent studies show that exosomes that transport specific microRNAs (miRNAs) are involved in tumor development. However, the molecular mechanism by which tumor invasion and migration are regulated by exosomes from non-small cell lung cancer (NSCLC) is not well understood. Here, we show that exosomes shuttling low levels of miR-34c-3p are involved in NSCLC progression. Our results showed that exosomes derived from NSCLC cells carrying low levels of miR-34c-3p could be transported into the cytoplasm of NSCLC cells and accelerate NSCLC invasion and migration by upregulating integrin α2β1. A luciferase assay revealed that integrin α2β1 was the direct target of miR-34c-3p, and overexpression of integrin α2β1 could promote the invasion and migration of NSCLC cells. The analysis of exosomes derived from clinical serum samples indicated that the expression of miR-34c-3p was significantly downregulated in exosomes from NSCLC patients compared with that of normal controls. A549-derived exosomes promoted NSCLC cells lung metastases in vivo. Exosomes shuttling low levels of miR-34c-3p were associated with the progression of NSCLC in vitro and in vivo. Our data demonstrate that exosomes shuttling low levels of miR-34c-3p can accelerate the invasion and migration of NSCLC by upregulating integrin α2β1. MiR-34c-3p can be a diagnostic and prognostic marker for NSCLC. High expression of integrin α2β1 is positively related to the migration and metastasis of NSCLC cells.

## Introduction

It is known that lung cancer plays is responsible for a large number of cancer-related deaths worldwide.^1^ Although there have been great improvements in both diagnosis and treatment, the mortality of lung cancer remains high. The 5-year survival of lung cancer is below 15%.^2^ Lung cancer is usually classified as non-small cell lung cancer (NSCLC) or small cell lung cancer (SCLC). NSCLC is more common, and it more easily metastasizes.^3^ Understanding the molecular mechanisms involved in the development of NSCLC will help in prognosis and in the development of novel therapeutic targets.^4^

Exosomes are endosome-derived vesicles (30–120 nm in size) formed in the vesicular bodies of the endosomal network. They serve an essential function in cellular communication.^5^ Exosomes are involved not only in cell–cell communication in the tumor microenvironment but also between donor and recipient cells, where they support the secretion of cytokines, growth factors, angiopoietin, and subsequent induction of proliferation, invasion and metastasis of recipient cells.^6,7^ Cancer-derived exosomes contain a wide range of components, such as lipids, proteins, DNAs, mRNAs and microRNAs (miRNAs). Experimental evidence indicates that miRNAs can be transferred between cells by exosomes.^8,9^

miRNAs are endogenous ~23 nt RNAs that play vital roles in gene regulation in plants or animals. MiRNAs interact with the mRNAs of protein-coding genes to repress gene expression at a posttranscriptional level.^10–12^ Recent studies revealed that miR-34c-3p promoted the growth of glioma cells, and a decrease in miR-34c-3p enabled glioma tumor-initiating cells to maintain self-renewal characteristics and resulted in antiapoptotic effects.^13^ In this article, exosomes were derived from NSCLC cells, and their involvement in the promotion of migration and invasion were investigated; further, there was investigation into the function of the miRNAs (such as miR-34c-3p) that they contained and the mechanisms in which they were involved.

## Results

### Characterization and uptake of exosomes

**Fig. 1:**
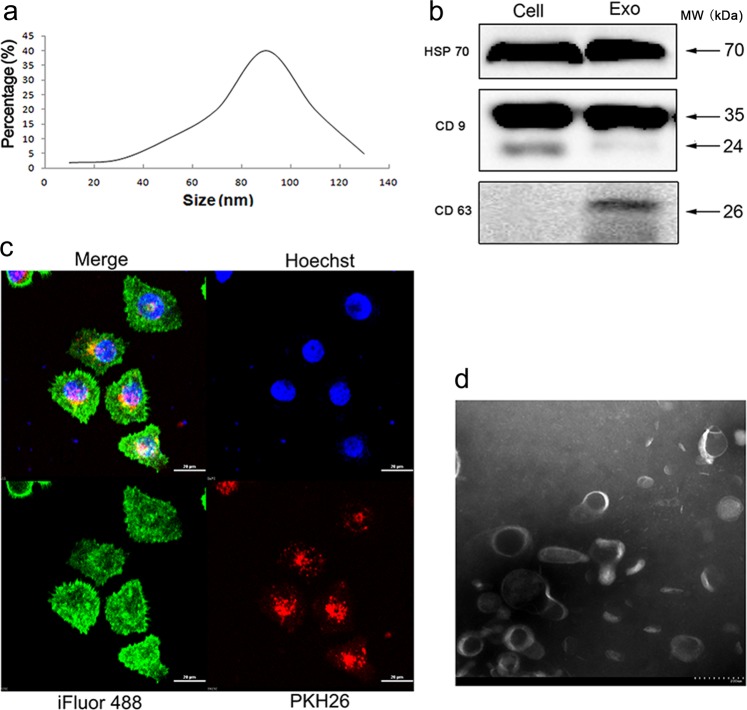
Characterization and uptake of exosomes. **a** A549-derived exosomes (A-exo) were determined to be between 30 and 120 nm in size by nanoparticle tracking analysis. **b** Western blots for HSP70, CD9, and CD63 in exosomes and cells. **c** Confocal microscopy of A549 cells treated with A549 exosomes labeled with the fluorescent linker PKH26 (red). The cytoskeleton of A549 cells was labeled with iFlour 488 Reagent (green), and the nucleus of A549 cells was labeled with Hoechst (blue). **d** Transmission electron microscopy image of exosomes are shown

Exosomes are small vesicles formed by membranous phospholipid bilayers. They range from 30 to 120 nm in diameter and have various biological and pathological functions that relate to tumor progression. To explore the effects of NSCLC-derived exosomes on tumor invasion and metastasis, we isolated exosomes from the supernatant of NSCLC cells using differential centrifugation. To confirm that the material we isolated was indeed exosomes, we used several methods according to the instructions provided in the Minimal information for studies of extracellular vesicles 2018 (MISEV2018).^14^ First, nanoparticle tracking analysis was used to examine the size of the exosomes. We found that exosomes derived from NSCLC cells were round vesicles that ranged from 30 to 120 nm in size (Fig. 1a). Second, Western blots were applied to characterize the protein composition of the NSCLC cell exosomes. As shown in Fig. 1b, exosome markers CD9 and CD63 were abundant in our exosome preparations. To confirm the ability of NSCLC cells to uptake exosomes, recipient cells were cultured with PKH-26-labeled exosomes for 12 h (×600, Fig. 1c). The results showed that exosomes were taken up and were transported into the cytoplasm of recipient cells. In addition, the exosome preparation was confirmed to contain round vesicles measuring 30–120 nm in diameter by electron microscopy (Fig. 1d). These results indicated that the exosomes isolated from NSCLC cells were sufficiently pure for subsequent experiments.

### Exosomes from NSCLC cells induced cell invasion and migration

**Fig. 2:**
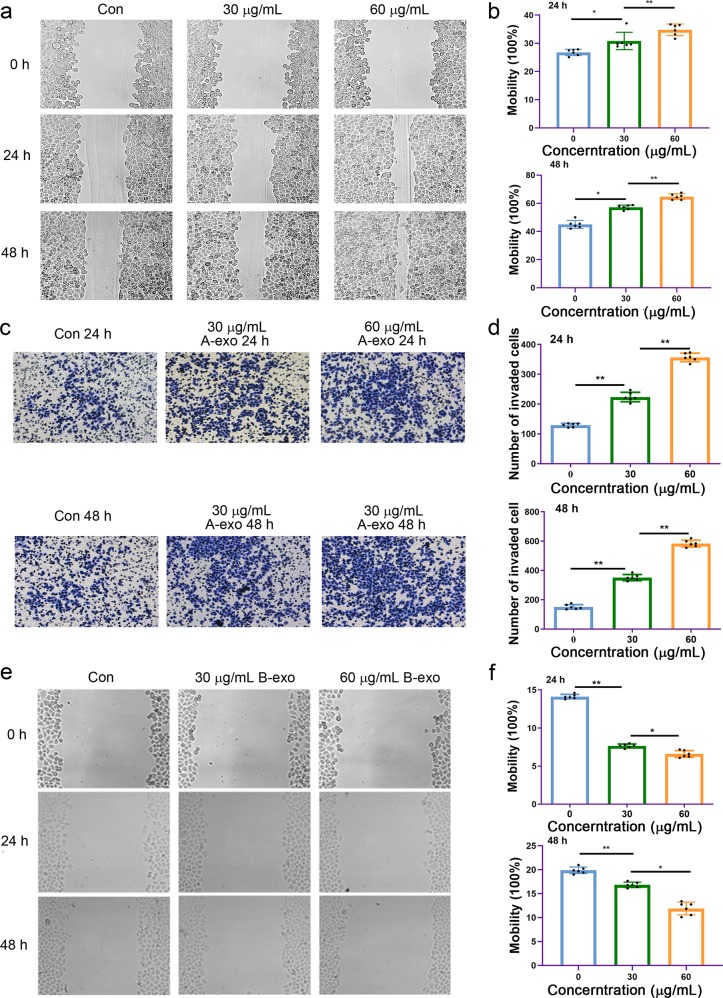
Increased invasion and migration of A549 cells following treatment with exosomes derived from NSCLC. **a** NSCLC A549 cell migration was induced by exosomes derived from A549 cells (A-exo) in a dose- and time-dependent manner. **b** Quantitative results of **a**. ns no significance, *p < 0.05, **p < 0.01, and *n* ≥ 3. **c** A549 cell invasion was induced by A549 cell-derived exosomes (A-exo) in a dose- and time-dependent manner. **d** Quantitative results of **c**. *p < 0.05, **p < 0.01, and *n* ≥ 3. **e** A549 cell migration was inhibited by BEAS-2B cell-derived exosomes (B-exo) in a dose-dependent manner. **f** Quantitative results of **e**. ns no significance, *p < 0.05, **p < 0.01, and *n* ≥ 3

Tumor cells secrete a large number of exosomes that promote tumor invasion and migration by mediating cellular communication between tumor cells and the surrounding stromal tissue. Exosomes from tumor cells promote the activation of proliferative and angiogenic pathways.^15^ To observe the activity of NSCLC-derived exosomes, A549 and PC-9 cells were treated with their own exosomes in doses of 30 and 60 μg/mL of exosomal protein, and changes in cell proliferation, invasion and migration were monitored. The results showed that NSCLC exosomes significantly increased A549 and PC-9 cell invasion and migration in a dose- and time-dependent manner (Fig. 2a, b for migration ability, 2c and d for invasion ability) (Fig. 2 for A549 cells, Supplementary Data Fig. S2 for PC-9 cells). On the other hand, exosomes derived from BEAS-2B cells inhibited the migration and invasion of A549 cells (Fig. 2e, f).

### The miRNA expression profile of NSCLC-derived exosomes

**Fig. 3:**
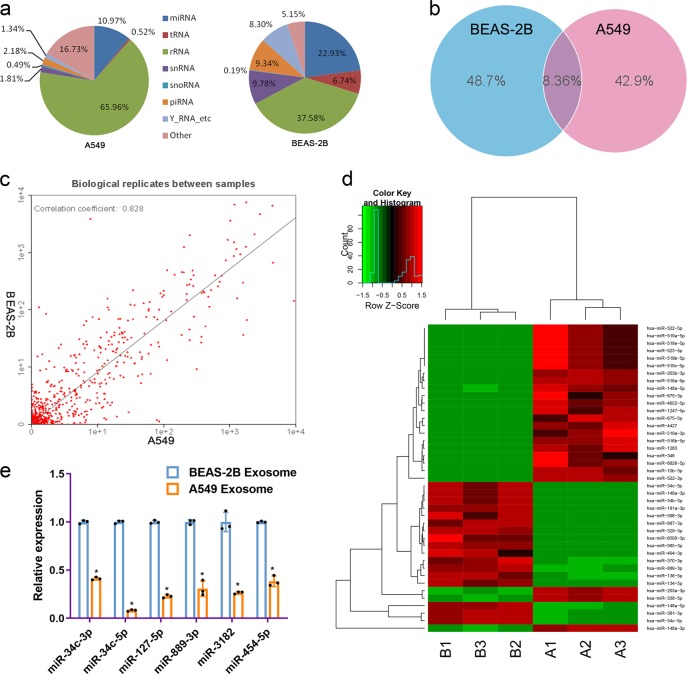
Profiles of miRNA expression in exosomes derived from different cells. **a** The ratio of the percentage small RNA categories in all reads mapped to noncoding RNA databases. **b** Venn diagram showing the unique and overlapping miRNAs between exosomes from different cells. **c** Biological replicates between exosomes from BEAS-2B and A549 cells. **d** Heatmap diagram of differential miRNA expression between exosomes derived from BEAS-2B cells and A549 cells. Gene expression data were obtained using next-generation sequencing on an Illumina HiSeq 2500 platform. Mean expression values are shown. Red, increased expression; green, decreased expression; and black, mean value. **p < 0.01. **e** Determination of miR-34c-3p, miR-34c-5p, miR-127-5p, miR-889-3p, miR-3182 and miR-454-5p microRNA levels by quantitative real-time PCR in exosomes from NSCLC cells. Experiments were performed in triplicate

MiRNA expression patterns vary by cell type. The miRNA expression profiles of exosomes derived from NSCLC cells were investigated using Illumina HiSeq 2500 high-throughput sequencing (miRNA-seq). The percentage of miRNAs in the total RNA isolated from exosomes from human lung cancer A549 cells or human bronchial epithelial BEAS-2B cells was 10.97% and 22.93%, respectively (Fig. 3a).

To distinguish between the different miRNAs, we compared all ncRNA reads from the exosome libraries of known human miRNAs in miRBase v20. We removed the ncRNA sequences with less than 10 reads and focused on the well-represented miRNAs. Finally, we identified an average of 761 and 800 types of known miRNAs in normal and NSCLC exosomes, respectively. Additionally, 8.36% of the miRNAs were shared between the exosomes derived from A549 and BEAS-2B cells. The number of unique and overlapping miRNAs contained in exosomes isolated from the two different cell lines is shown in Fig. 3b. A 2-fold change and **p < 0.01 were the threshold cutoffs used to compare the expression of miRNAs in exosomes from the two cell lines. It was found that 317 miRNAs exhibited significant differences between BEAS-2B cells and A549 cell-derived exosomes. Selected data are displayed in Fig. 3e. The correlation coefficient between exosomes derived from BEAS-2B and A549 cells was 0.828 (Fig. 3c). To validate the results of miRNA sequencing, we used quantitative real-time PCR to measure the expression of some of the identified miRNAs. The levels of miR-34c-3p, miR-34c-5p, miR-127-5p, miR-889-3p, miR-3182, and miR-454-5p in exosomes derived from A549 cells were significantly lower than those in exosomes from BEAS-2B cells (Fig. 3e), which was consistent with the miRNA sequencing results (Fig. 3d, Table 1).

**Table 1:** Differential expression of miRNAs in exosomes from BEAS-2B cells (B) and A549 cells (A) by sequencing

Systematic name of miRNA	Expression level of A compared with B	Fold change (A/B)	*P*-value
hsa-miR-146a-5p	Down	9.1288	0.0000
hsa-miR-203a-3p	Up	9.4194	0.0000
hsa-miR-338-5p	Up	8.6978	0.0000
hsa-miR-381-3p	Down	6.9464	0.0000
hsa-miR-34c-5p	Down	8.468	0.0000
hsa-miR-148a-3p	Up	6.0519	0.0000
hsa-miR-522-3p	Up	9.8726	0.0000
hsa-miR-134-5p	Down	9.4913	0.0000
hsa-miR-1247-5p	Up	9.0756	0.0000
hsa-miR-370-3p	Down	7.007	0.0000
hsa-miR-889-3p	Down	6.7839	0.0000
hsa-miR-203b-3p	Up	8.6302	0.0000
hsa-miR-4652-5p	Up	8.5325	0.0000
hsa-miR-138-5p	Down	6.5344	0.0000
hsa-miR-516a-5p	Up	8.4506	0.0000
hsa-miR-148a-5p	Up	6.1443	0.0000
hsa-miR-675-3p	Up	8.9271	0.0000
hsa-miR-519a-5p	Up	8.0025	0.0000
hsa-miR-522-5p	Up	8.0025	0.0000
hsa-miR-3182	Down	5.7591	0.0000
hsa-miR-127-5p	Down	4.4146	0.0000
hsa-miR-454-5p	Down	4.2333	0.0000
hsa-miR-519a-3p	Up	8.3696	0.0000
hsa-miR-34c-3p	Down	7.0425	0.0000

### Exosomal miR-34c-3p was depleted in the sera of patients with NSCLC

**Fig. 4:**
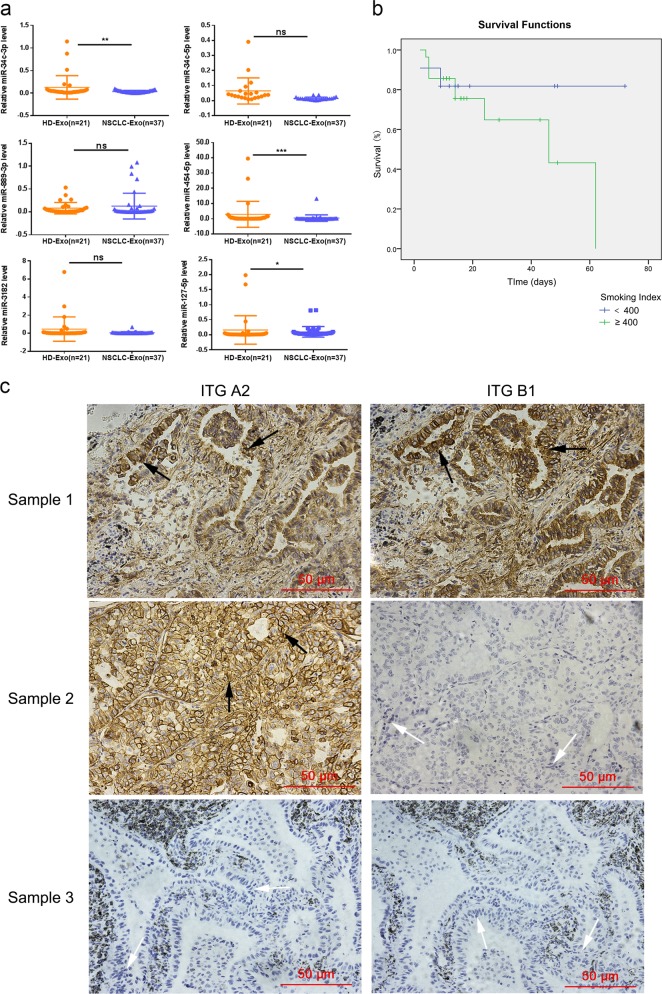
Integrin α2β1 protein and exosomal miRNA expression in clinical samples. **a** Levels of miRNAs in the sera of NSCLC patients and healthy controls. The levels of exosomal miR-34c-3p, miR-34c-5p, miR-889-3p, miR-454-5p, miR-3182, and miR-127-5p in the sera of NSCLC patients (*n* = 37) and healthy controls (*n* = 21) were measured by RT-qPCR. Differences in miRNA expression between NSCLC patients and healthy controls were analyzed by nonparametric test, ns no significance, *p < 0.05, **p < 0.01, and ***p < 0.001. **b** Kaplan-Meier curve analysis of NSCLC patients (*n* = 37) was performed according to the smoking index. **c** The protein expression of integrin α2 and β1 in NSCLC tissue samples (*n* = 50). Immunohistochemistry on the expression of integrin α2 and β1 in 50 human lung adenocarcinoma samples. Three representative staining images are shown, where either integrin α2 or integrin β1 staining was positive (white arrow) or negative (black arrow). ITG A2 Integrin α2, and ITG B1 Integrin β1

We found that the level of exosomal miR-34c-3p was significantly lower in the serum of NSCLC patients (*n* = 37) than it was in healthy controls (*n* = 21). Combining this finding with the results of the miRNA library sequencing, we concluded that the levels of exosomal miR-34c-3p, miR-454-5p, and miR-127-5p were significantly lower in patients with NSCLC than they were in control patients (Fig. 4a). Moreover, the levels of miR-34c-3p and miR-454-5p showed a potential correlation with the incidence of NSCLC (*p* = 0.033 and *p* = 0.045, respectively). Fig. 4b shows a Kaplan-Meier curve of NSCLC patients, which finds that heavy addiction to smoking leads to a poor prognosis for NSCLC.

### Integrin α2 and integrin β1 were highly expressed in human NSCLC tissues

It is well known that overexpression of integrin is closely associated with tumorigenesis and progression of disease.^16^ In this study, immunohistochemistry (IHC) analysis was used to examine the expression of integrin α2 and β1 in NSCLC tissues (Fig. 4c shows representative images). Table 2 shows that integrin α2 was overexpressed in 49 out of 50 cases (98.0% positive expression rate); integrin β1 was also increased in human NSCLC tissues, with 38.0% of patients exhibiting positive expression. These findings suggested that integrin α2β1 is an important oncogene in NSCLC. The scoring was based on both the intensity of staining and the number of positively stained cells. The intensity of the dye color (a) was graded as 0 (no color), 1 (light yellow), 2 (light brown), or 3 (brown). The number of positive cells (b) was classified as 0 (1–10%), 1 (11–50%), 2 (25–50%), 3 (51–80%), or 4 (>80%). The values for (a) and (b) were multiplied, and specimens were assigned to one of the four levels: 0 score: (−), 1–4 score: (+), 5–8 score: (++), more than 8 score: (+++). Statistical analyses of gender, age, stage, and histological grade are shown in Table 2.

**Table 2:** Integrin α2 and Integrin β1 expression with clinicopathological variables in 50 NSCLC samples

		*n*	Integrin α2	*P*	Integrin β1	*P*
Age (years)	≥60	29	28	0.577^b^	9	0.233^a^
	<60	21	21		10	
Gender	Male	33	33	0.340^b^	12	0.740^a^
	Female	17	16		7	
Histological Grade	Well-differentiate	3	2	0.060^b^	1	1.000^b^
	Moderately-differentiate	27	27		10	
	Poor-differentiate	20	20		8	
Stage	0	1	0	0.020^b^	0	0.799^b^
	I	23	23		8	
	II	22	22		10	
	III	4	4		1	

^a^Continuity correction Chi-square

^b^Fisher’s Exact Test

### Exosomes from NSCLC cells promoted integrin α2β1 expression in NSCLC cells

**Fig. 5:**
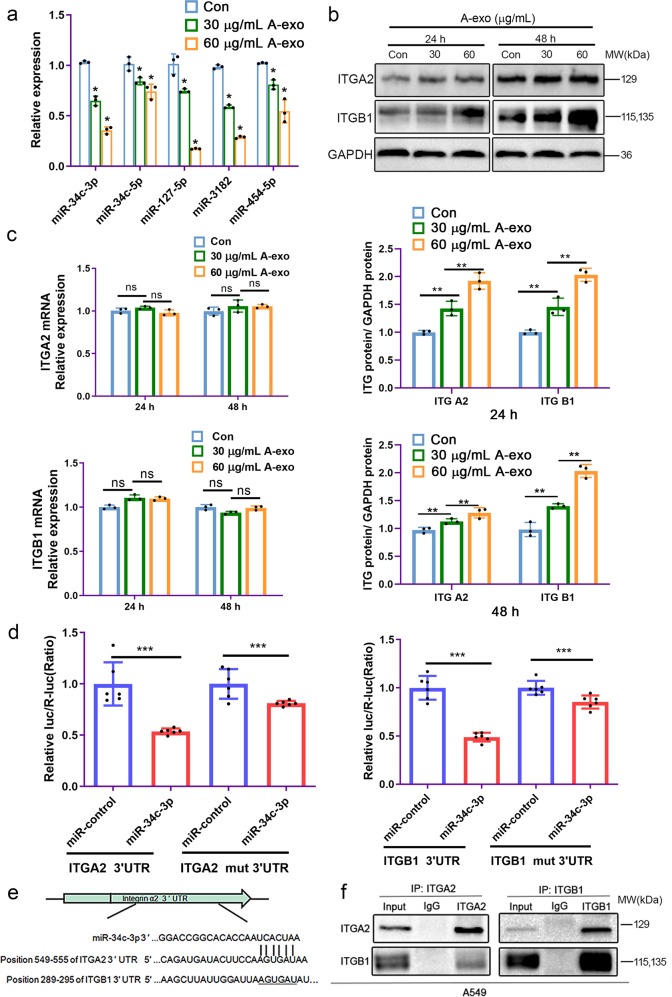
NSCLC exosomes increased the protein levels of integrin α2β1 by reducing miR-34c-3p. **a** NSCLC exosomes reduced the expression of miR-34c-3p. Cellular miR-34c-3p levels were detected by quantitative RT-PCR after treatment with NSCLC exosomes. **b** NSCLC exosomes increased the protein levels of integrin α2β1 in NSCLC cells. Western blotting was performed with the indicated antibodies, and GAPDH was used as a control to normalize levels. **c** NSCLC exosomes did not affect the mRNA levels of integrin α2β1 in NSCLC cells. The mRNA levels of integrin α2β1 in cells were detected by quantitative RT-PCR after treatment with NSCLC exosomes. **d** Luciferase activity by luciferase reporters carrying the 3′-UTR of ITGA2 or ITGB1 or mutant ITGA2 or ITGB1 in which the binding site for miR-34c-3p was mutated. The vectors were introduced into 293 T cells along with a negative miR-control (NC) or miR-34c-3p. **e** Alignment of ITGA2 and ITGB1 3′-UTRs with miR-34c-3p. f The results of coimmunoprecipitation reflect the fact that integrin α2 interacts with integrin β1 in NSCLC cells. ns no significance, *p < 0.05, **p < 0.01, ***p < 0.001, and *n* ≥ 3. ITG A2 Integrin α2, and ITG B1 Integrin β1

As described above, exosomes from NSCLC cells promoted cell invasion and migration. Additionally, the relative expression of several microRNAs (miR-34c-3p, miR-34c-5p, miR-127-5p, miR-3182, and miR-454-5p) in exosomes derived from NSCLC A549 cells was lower than it was in exosomes derived from bronchial epithelial BEAS-2B cells (Fig. 3e). To confirm the role of NSCLC exosomes in promoting invasion and metastasis, we examined the expression of miR-34c-3p and integrin α2β1 in A549 cells after various treatments with A549 exosomes. Compared with the control group, miR-34c-3p levels were markedly decreased after treatment with different concentrations of NSCLC exosomes (Fig. 5a), while integrin α2β1 protein expression was upregulated, especially after 48 h of treatment (Fig. 5b). However, the addition of NSCLC exosomes did not affect the integrin α2β1 mRNA levels (Fig. 5c).

We also examined integrin α2β1 protein levels in cells from different groups and found that the administration of BEAS-2B-derived exosomes downregulated the expression of integrin α2β1 (Supplementary Fig. S1).

### miR-34c-3p directly targeted integrin α2β1

Using TargetScan software for prediction, we performed sequence alignment, which identified potential binding sites for miR-34c-3p in the 3′-UTRs of integrin α2 (ITGA2) and integrin β1 (ITGB1) mRNAs (Fig. 5e), suggesting that miR-34c-3p might regulate the expression of ITGA2 and ITGB1. The relative luc/R-luc signal was 53.74 ± 3.63% and 81.03 ± 8.68% in the miR-34c-3p group cotransfected with the ITGA2 3′-UTR and mut 3′-UTR reporters, respectively. The relative luc/R-luc signal was 48.82 ± 7.88% and 85.28 ± 6.78% in the miR-34c-3p group cotransfected with the ITGB1 3′-UTR and mut 3′-UTR reporters, respectively. The results indicated that the relative luc/R-luc signal in the presence of miR-34c-3p was restored after a mutation had been introduced at the miR-34c-3p binding site (Fig. 5d). The results of coimmunoprecipitation (Fig. 5f) reflect the interaction between integrin α2 and β1 in NSCLC cells, as expected.

### Inhibition of miR-34c-3p upregulated the level of integrin α2β1 and promoted tumor cell migration

**Fig. 6:**
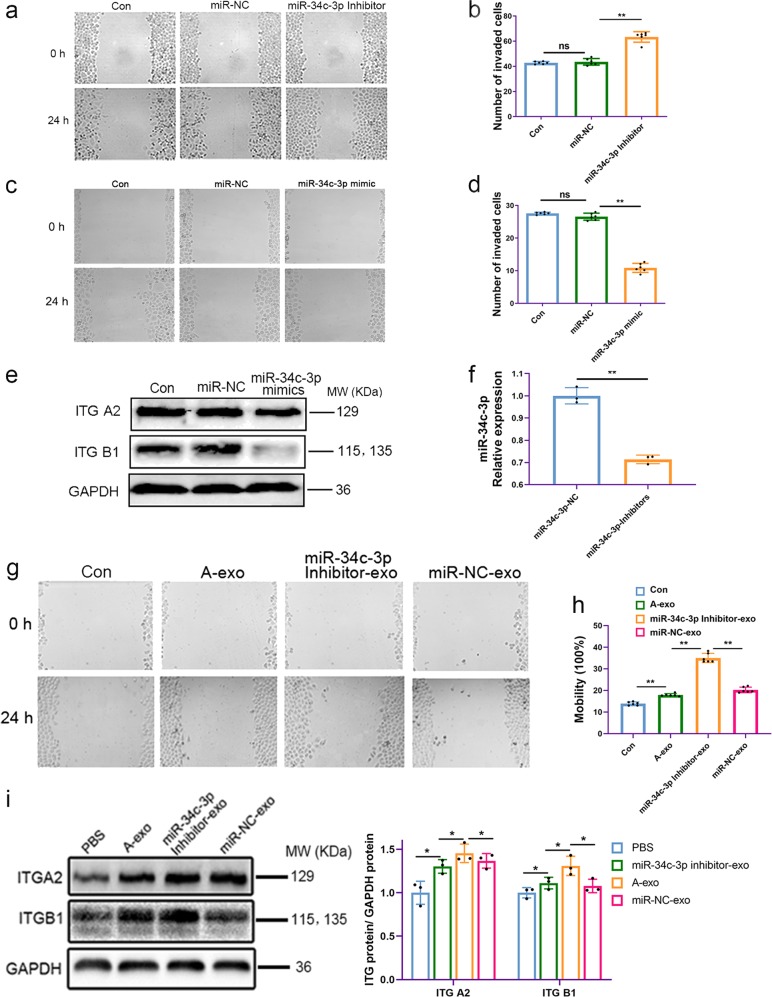
Inhibition of miR-34c-3p in exosomes induced A549 cell migration by regulating integrin α2β1. **a** Migration of A549 cells was induced by transfection with miR-34c-3p inhibitor. **b** Data analysis of **a**. **c** A549 cell migration was inhibited by transfection with miR-34c-3p mimics. **d** Data analysis of **c**. ns no significance, *p < 0.05, **p < 0.01, and *n* ≥ 3. **e** Western blot of integrin α2β1 expression in A549 cells after transfection with miR-34c-3p mimics. **f** Reduction in the level of miR-34c-3p in exosomes upon transfection with a miR-34c-3p inhibitor in A549 cells. **g** Induction of A549 cell migration by miR-34c-3p inhibitor-exo. **h** Data analysis of Fig. 6g. **i** Western blot analysis of integrin α2β1 expression from different groups, and GAPDH was used as a control to normalize levels. ns no significance, *p < 0.05, **p < 0.01, ***p < 0.001, and *n* ≥ 3. ITG A2 Integrin α2, and ITG B1 Integrin β1

To determine the relationship between the expression of miR-34c-3p and tumor cell migration, NSCLC cells were transfected with miR-34c-3p mimics or a miR-34c-3p inhibitor (as described in Methods). Administration of the miR-34c-3p inhibitor promoted cell migration, while miR-34c-3p mimics inhibited the invasion and migration of NSCLC cells (Fig. 6a–d).

The effects of NSCLC exosomes pretreated with a miR-34c-3p inhibitor were then compared with those of untreated NSCLC exosomes in terms of invasion and metastasis. After miR-34c-3p mimics were transfected into tumor cells, the protein levels of integrin α2β1 decreased relative to those of miR-NC-transfected tumor cells (Fig. 6e). The level of miR-34c-3p in exosomes decreased in A549 cells transfected with a miR-34c-3p inhibitor (Fig. 6f). Additionally, miR-34c-3p inhibitor-exosome administration dramatically promoted cell migration in A549 cells after 24 h compared to cells treatment with NSCLC exosomes (Fig. 6g, h). Administration of miR-34c-3p inhibitor exosomes significantly increased the protein levels of integrin α2β1 after 24 h compared to what was observed following NSCLC exosome administration (Fig. 6i). These results indicated that miR-34c-3p is involved in regulating tumor progression by modulating the expression of integrin α2β1 in NSCLC cells.

### A549-derived exosomes promoted NSCLC cells lung metastases in vivo

**Fig. 7:**
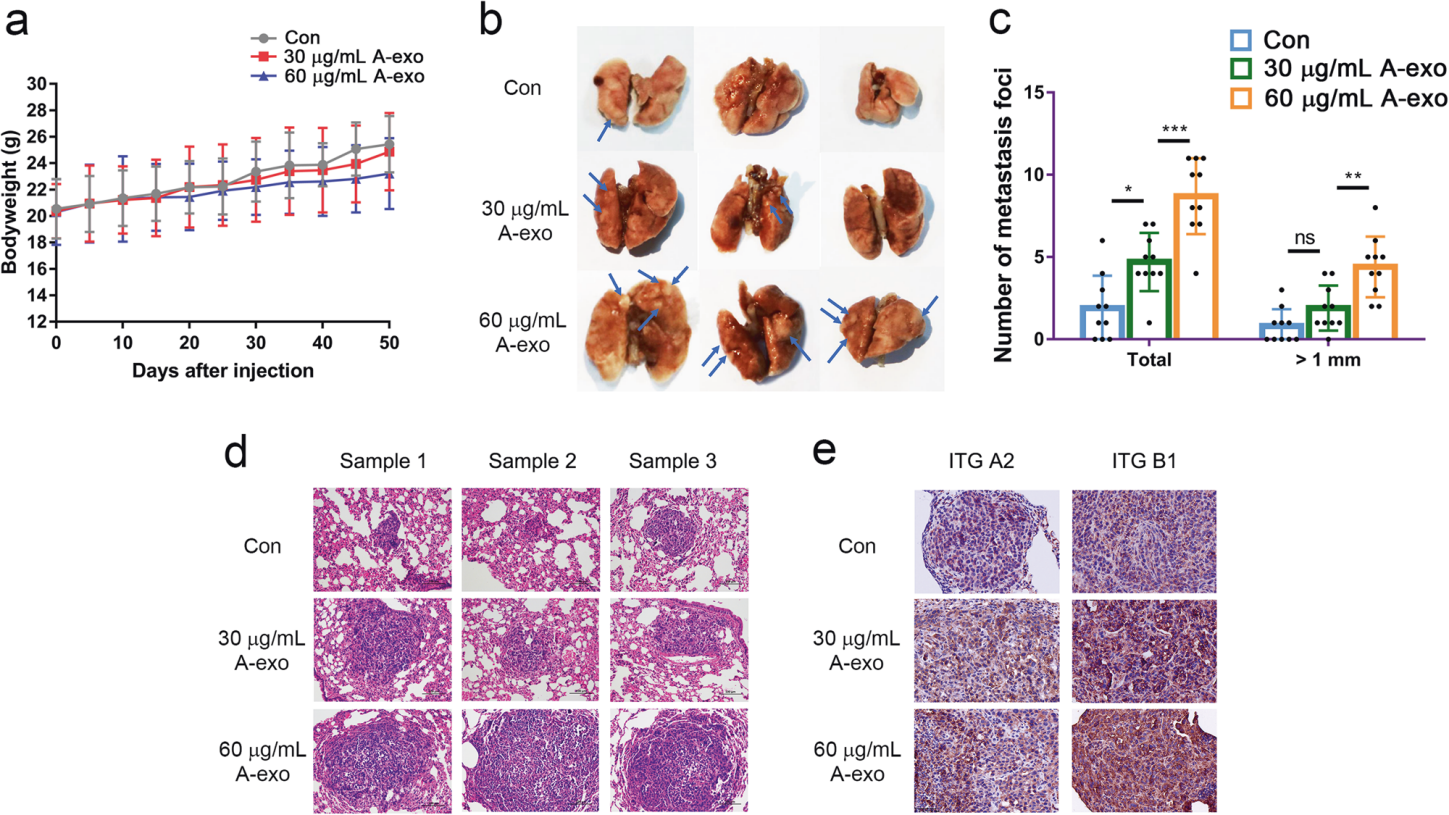
A549-derived exosomes promoted NSCLC cell metastasis in vivo. **a** A549-derived exosome treatment caused loss of bodyweight in mice. **b** Exosome-incubated A549 cells developed more or larger metastatic nodules in the lungs than untreated cells, and the effect was dose-dependent manner. **c** The statistical analysis of total metastatic foci and metastatic foci with diameters greater than 1 mm, ns no significance, *p < 0.05, **p < 0.01, and ***p < 0.001. **d** H&E staining of tumor specimens showed that the higher concentration of A549 exosomes caused increased malignant metastases (200×). **e** A549-derived exosomes promoted NSCLC cell metastasis in vivo in a dose-dependent manner, and the highly metastatic samples showed a higher expression of ITGA2 and ITGB1. ITG A2 Integrin α2, and ITG B1 Integrin β1 (400×)

To further investigate the role of A549 exosomes in vivo, we incubated A549 cells with A549-derived exosomes at 30 μg/mL and 60 μg/mL for 24 h. Then, exosome-treated and untreated A549 cells were injected (i.v.) into BALB/c nude mice via the tail vein. Bodyweight was measured periodically. Seven weeks post-inoculation, the mice were euthanized, and their lungs were removed to evaluate metastases and histology. The results showed that exosome treatment caused bodyweight loss in mice (*p* > 0.05) (Fig. 7a). Importantly, the mice injected with exosome-incubated A549 cells developed more or larger metastatic nodules in the lungs compared with what was observed following injection with untreated cells, and the effect was dose-dependent (Fig. 7b, c). Consistently, H&E staining of the tumor specimens indicated that the higher concentration of A549 exosomes caused an increased number of malignant metastases (Fig. 7d). IHC was subsequently applied to detect the protein expression of ITGA2 and ITGB1. As expected, the metastases of the exosome-incubated group exhibited higher ITGA2 and ITGB1 expression than those of the untreated group (Fig. 7e). Taken together, the in vivo metastasis experiments confirmed that A549-derived exosomes promoted NSCLC cell metastasis in vivo in a dose-dependent manner, and they positively correlated with the expression of ITGA2 and ITGB1.

## Discussion

It was reported that exosomes containing miRNAs play pivotal roles in various devastating diseases and infections.^17^ There has been increasing interest in recent years in the use of exosomes as biomarkers or therapeutic targets, particularly in the field of oncology.^18^ Numerous studies on the role of exosomes in cancer progression have been published.^19,20^

During the developmental process of human diseases, the composition of exosomes becomes profoundly altered.^21^ These changes include alterations in the types and quantities of various miRNAs, suggesting a potential for exosomes to be used for drug delivery. In our study, we investigated the expression profiles of exosomal miRNAs derived from NSCLC cells using Illumina HiSeq 2500 high-throughput sequencing (miRNA-seq). The percentages of miRNAs contained in the total RNA isolated from exosomes from A549 and BEAS-2B cell was 10.97% and 22.93%, respectively. The miRNA-seq report was validated by quantitative real-time PCR. The results showed that the amount of miR-34c-3p, miR-34c-5p, miR-127-5p, miR-889-3p, miR-3182, and miR-454-5p in exosomes derived from A549 cells was significantly lower than that of miRNAs from BEAS-2B cells.

It is well known that exosomes have essential functions in cellular communication.^5^ This not only favors the secretion of growth factors and cytokines involved in cell–cell communication in the tumor microenvironment but also induces the proliferation, invasion, and metastasis of cancer cells.^17–20^ Our study also demonstrated that NSCLC exosomes promoted tumor cell invasion and metastasis (Fig. 2a–d and Supplementary Fig. S2b–e). We found that exosomes from A549 cells were taken up by A549 cells and induced a dramatic increase in the invasion and migration of NSCLC cells in a dose- and time-dependent manner, but exosomes from human bronchial epithelial BEAS-2B cells inhibited the migration of A549 cells. These findings indicate that exosomes isolated from NSCLC play an important role in promoting invasion and metastasis. The Hsa-miR-34 family is a highly conserved miRNA family that contains three members, hsa-miR-34a, hsa-miR-34b, and hsa-miR-34c. The three members have highly homologous sequences, so they have similar target genes and functions.^22^ The hsa-miR-34a gene is located at chromosome 1p36, hsa-miR-34b and c are cotranscribed because they are part of the same gene cluster, and their location is 11q23.^23^ A number of studies have found that hsa-miR-34c is downregulated in various tumors, such as breast cancer and prostate cancer, and it plays a role as a tumor suppressor gene.^24,25^ MiR-34c is expressed at low levels in NSCLC; overexpression of miR-34c can inhibit proliferation, induce apoptosis, and inhibit the invasion or metastasis of A549 cells.^26^ However, there are few reports on the clinical significance of miR-34c-3p expression in NSCLC-derived exosomes, and the molecular mechanism regulating the development of NSCLC has not been elucidated. In the present study, we observed that the level of miR-34c-3p in exosomes from the serum of NSCLC patients or from NSCLC cell lines (A549 and PC-9), was significantly lower than it was in exosomes from healthy patients or BEAS-2B cells (Fig. 3e, Fig. 4a and Supplementary Fig. S2a). More importantly, the level of miR-34c-3p in exosomes from the serum of NSCLC patients showed a significant negative association with disease-free survival (*p* < 0.05).

Integrins, formed from α and β subunits, are cell-surface adhesive receptors that mediate cell adhesion. Integrins may trigger a variety of signaling pathways and regulate cell survival, differentiation, and migration, including cytoplasmic alkalization, calcium influx, potassium channel activation, activation of MAP kinase cascades and tyrosine phosphorylation of regulatory proteins.^27,28^ The results of coimmunoprecipitation in our study verified that integrin α2 indeed interacts with integrin β1 in NSCLC cells (Fig. 5f). In our study, the overexpression of the integrin α2 subunit in human NSCLC tissues was detected by immunohistochemistry, with a positive expression rate of 98.0%. However, the positive rates of integrin α2 and integrin β1 in our IHC analysis were different. This might be because the number of samples from NSCLC was not large enough. Our results showed that the levels of integrin α2 or β1 did not depend on gender, age, or clinical stage of histological grade (Fig. 4c and Table 2).

To determine whether the positive effects of exosomes from NSCLC cells tumor on invasion and metastasis were mediated directly by exosome-delivered miR-34c-3p, the following experiments were performed in our study. We first examined the level of miR-34c-3p in cells in the presence or absence of exosomes. Our results indicated that the expression of endogenous miR-34c-3p decreased in NSCLC A549 cells treated with A549 cell exosomes (Fig. 5a). These results indicate that the decreased miR-34c-3p level in NSCLC cells could be attributed to the addition of NSCLC exosomes. The expression of miR-34c-3p in NSCLC A549 cells was downregulated and upregulated by treatment with a miR-34c-3p inhibitor and with mimics, respectively. NSCLC A549 cell migration was increased when the expression of endogenous or exogenous miR-34c-3p was blocked; conversely, migration was inhibited when the miR-34c-3p level was increased (Fig. 6a–d).

By what mechanism do exosomes carrying miR-34c-3p promote the invasion and metastasis of NSCLC A549 cells? To answer this question, TargetScan software prediction data was first analyzed, and it indicated that miR-34c-3p could bind to the 3′-UTRs of ITGA2 and ITGB1 mRNA. Next, luciferase assays were performed, and the results indicated that the relative luc/R-luc of miR-34c-3p was restored when a mutation at the predicted binding sites for miR-34c-3p in the 3′-UTR of the integrins was introduced, suggesting that integrin α2β1 was the direct downstream target of miR-34c-3p (Fig. 5d, e). Moreover, exosomes from NSCLC A549 cells upregulated the protein level of integrin α2β1 but did not affect integrin α2β1 mRNA expression (Fig. 5b, c), which suggested that miR-34c-3p regulates integrin α2β1 post-transcriptionally.

To explore the effect of A549-derived exosomes in vivo, A549 cells were cocultured with different concentrations of A549-derived exosomes and were subsequently injected (i.v.) into the tail vein of nude mice to establish a pulmonary metastasis model. After incubation with different concentrations of A549-derived exosomes, A549 cells caused increased metastatic characteristics, which caused a decrease in bodyweight (*p* > 0.05) (Fig. 7a), more or larger metastatic foci in the lungs (Fig. 7b and c), and more malignant metastases observed than those in control animals (Fig. 7d). Importantly, consistent with the in vitro results (Fig. 5b) and clinical samples (Table 2 and Fig. 4c), these highly metastatic samples showed higher expression of both ITGA2 and ITGB1 (Fig. 7e).

Furthermore, we demonstrated that exosomal miR-34c-3p was decreased after transfection of A549 cells with miR-34c-3p inhibitor (Fig. 6f). We further revealed that downregulation of miR-34c-3p mediated by exosomes accelerated cell migration (Fig. 6g, h) and increased the protein levels of integrin α2β1 by treatment with miR-34c-3p inhibitor-exo (Fig. 6i). Here, we are the first to propose that exosomes shuttling low levels of miR-34c-3p can accelerate invasion and metastasis of NSCLC cells via upregulating integrin α2β1.

In summary, our study elucidates that low levels of miR-34c-3p in exosomes promote NSCLC progression. Delivery of NSCLC exosomes with low levels of miR-34c-3p to recipient cells subsequently increase the level of integrin α2β1 and accelerate invasion and metastasis of NSCLC cells in vitro and in vivo. Therefore, we suggest that low levels of miR-34c-3p in exosomes might serve as a potential therapeutic target and prognostic marker for clinical therapy in NSCLC patients. High expression of integrin α2β1 is positively related to the migration and metastasis of NSCLC cells.

## Materials and methods

### Cell culture

The human lung adenocarcinoma A549 cell line and the human bronchial epithelial BEAS-2B cell line that have acquired short tandem repeats (STRs) were purchased from iCell Bioscience Inc., Shanghai, China. The human lung adenocarcinoma cell line PC-9 (carrying the delE746-A750 mutation in the EGFR gene) was kindly provided by Professor Li-wu Fu (Sun Yat-sen University). A549 and PC-9 cells were cultured in RMPI 1640 medium, and BEAS-2B cells were cultured in DMEM medium. Both cultures were maintained at 37 °C in a 5% CO_2_ atmosphere. Both media contained 10% exosome-depleted fetal bovine serum (EXO-FBS-50A-1, System Biosciences, Palo Alto, CA) and 1% penicillin-streptomycin (Tianhang Biotechnology, Hangzhou, China).^29^

### Preparation of NSCLC tissue samples

Human lung cancer tissues were collected from 50 patients admitted to Shanxi Provincial People’s Hospital between 2011 and 2014. The patients’ ages ranged from 42 to 81 years. There were 34 males and 16 females. Patients received no chemotherapy before surgery. The cancer tissue samples were subsequently fixed in PBS containing 4% paraformaldehyde and then were embedded in paraffin for immunohistochemistry experiments. All participants provided written informed consent. This study followed the ethical guidelines and was approved by the Ethics Committee.

### Clinical serum samples

We collected serum samples from 21 healthy persons and 37 patients at DaTong Second People’s Hospital. All participants provided written informed consent before inclusion in the study. The study followed the ethical guidelines and was approved by the Ethics Committee of DaTong Second People’s Hospital.

### Exosome isolation and identification

Differential centrifugation was used to isolate exosomes as previously described.^30^ The isolated exosomes were visualized by transmission electron microscopy (TEM) according to methods described by Gurunathan et al.^31^ Briefly, exosomes to be examined by TEM were resuspended in PBS buffer, dropped onto a carbon-coated copper grid, and were subsequently stained with 2% uranyl acetate. Images were taken by a JEM-1400 TEM (JEOL Ltd., Japan, located in Guangdong Institute of Microbiology, Guangzhou, China) operated at 80.0 kV.

The size and number of exosomes were directly monitored by the Nanosight NS 300 system (Nanosight Technology, Malvern, UK), which was assembled with a 488 nm laser and a high-sensitivity sCMOS camera.^32^ In detail, exosomes were resuspended at a concentration of 5 µg of protein/mL in phosphorous buffered saline (PBS), and they were subsequently diluted 100–500-fold to achieve between 20–100 particles per frame. Samples were manually loaded into the sample chamber at ambient temperature. Each sample was examined in triplicate at a camera setting of 13, with an acquisition time of 30 s and a detection threshold of 7. At least 200 completed tracks were analyzed per video. The data were captured and analyzed by NTA analytical software version 2.3.

### Exosome labeling and uptake by cells

Exosomes collected by differential centrifugation were incubated with PKH26 (Sigma, St. Louis, MO) for 5 min at room temperature. The labeled exosomes were washed with PBS by ultracentrifugation. The pellets were resuspended in low serum medium, and the suspension was incubated with A549 cells for 12 h. Cells that had been grown on coverslips (NEST Biotechnology, Wuxi, China) were stained with CytoPainter Phalloidin iFluor 488 Reagent (ab176753, Abcam), which binds to actin with high affinity. Nuclei were stained with Hoechst 33342 (Cell Signaling Technology, Danvers, MA). Exosomes taken up by cells were examined by a confocal microscope (×600).^33^

### Wound healing assay

Scratch assays were performed to determine cell migration ability in vitro as previously described.^34^ In brief, the cells were plated in 6-well plates and grown overnight so that they reached confluence. A 10 μL pipette tip was subsequently used to scratch the cell monolayer, and the wounded cell layer was washed to discard dead cells. The cells were incubated with exosomes (30 or 60 μg/mL of protein) for 0, 24, or 48 h. Images were captured under a microscope at ×200 magnification. Cell motility was defined as the percentage of the repaired area, i.e., percent wound closure (%) = migrated cell-surface area/total surface area × 100.

### Invasion assay

Cells were seeded in the upper chamber of the transwell apparatus (Greiner Bio-One, Frickenhausen, Germany), the bottom of which was coated with Matrigel (Corning Costar, Corning, NY). Then, exosomes (30 or 60 μg/mL) were added to the bottom chamber to act a chemoattractant. Cells that migrated to the lower chamber were fixed with methanol and were stained with 0.05% crystal violet. After cells in the top layer were wiped away with a cotton swab, the migrated cells were imaged. Nine random fields were photographed for quantification of migrated cells. Images were captured under a microscope at ×200 magnification. IMAGE-J software (http://imagej.nih.gov/ij/) was used to quantify the numbers of cells. The ratio of cells that migrated to the bottom layer and the total number of cells attached to the filter was calculated for each group.^35^

### Western blotting

Cultured cells were collected and lysed in lysis buffer containing a proteinase inhibitor cocktail and phenylmethylsulfonyl fluoride (PMSF, Cell Signaling Technology, Danvers, MA, USA). Equal amounts of proteins were separated on 8–12% sodium dodecyl sulfate polyacrylamide gels (SDS-PAGE) and then were transferred to PVDF membranes (Millipore, Boston, MA). The membranes were then blocked with 5% nonfat milk for 2 h at room temperature and incubated with primary antibodies overnight at 4 °C. After that, blots were incubated with horseradish peroxidase (HRP)-conjugated secondary antibodies and were visualized with enhanced chemiluminescence as previously described.^36^ The antibodies used in this study were as follows: rabbit anti-integrin alpha 2 (ab133557, Abcam), rabbit anti-integrin beta 1 (#9699, Cell Signaling Technology), mouse anti-GAPDH (MB001, Bioworld Technology), rabbit anti-HSP70 (BS6446, Bioworld Technology), rabbit anti-CD9 (#13174, Cell Signaling Technology), mouse anti-CD63 (ab193349, Abcam), rabbit ant-Tsg 101 mAb (E303, Bioworld Technology), HRP-conjugated goat anti-rabbit antibody (#7074, Cell Signaling Technology), and HRP-conjugated goat anti-mouse antibody (BS12478, Bioworld Technology).

### Transfection

The miR-34c-3p inhibitor, mimic, negative controls (miR-NC), and transfection kit were obtained from RiboBio (RiboFECT CP, Guangzhou, China). Transfection was carried out according to the manufacturer’s instructions. Based on the recommendation of the protocol, the cells were maintained in the transfection medium for 24 h to achieve high transfection efficiency.^37^

### miRNA extraction, reverse transcription, and RT-qPCR

After A549 cells were stimulated with exosomes (30 and 60 µg/mL) for 24 h, total RNA was extracted using TRIzol (Thermo, MA, USA). RNA from exosomes was separated and enriched by a Total Exosome RNA Isolation kit (System Biosciences, Palo Alto, CA) following the users’ guide. A Mir-X™ miRNA RT-qPCR SYBR kit (TaKaRa, Japan) was used to convert miRNAs into cDNA, and RT-qPCR was carried out according to the manufacturer’s protocol. The data were analyzed using the 2^−ΔΔCT^ method.^38^ The sequences of the primers used are listed in the Supplementary Material.

### Construction and sequencing of miRNA libraries

For the preparation and sequencing of miRNA libraries, total RNA from exosomes from A549 and BEAS-2B cells was extracted. Briefly, the total RNA samples were fractionated using a 15% Tris-borate-EDTA (TBE) polyacrylamide gel (Invitrogen), and small RNAs of 18–30 nt were applied for library preparation. Small RNAs were reverse transcribed and amplified using PCR. The PCR products were subjected to sequencing via the Illumina HiSeq 2500 platform (RiboBio, Guangzhou, China).^39^

### Prediction of miRNA target sites

TargetScan (http://www.targetscan.org/), PicTar (http://pictar.mdc-berlin.de/), and miRanda (http://www.microrna.org/) were used to predict the possible targets of hsa-miR-34c-3p. It was found that hsa-miR-34c-3p might target the 3′-UTR of integrin α2β1. The predicted target region occurs from nucleotides 549–555 in the integrin α2 (ITGA2) 3′-UTR (5′ CAGAUGAUACUUCCAAGUGAUAA) and from nucleotides 289–295 in the integrin β1 (ITGB1) 3′-UTR (5′ AAGCUUAUUGGAUUAAGUGAUAU); the miR-34c-3p sequence was GGACCGGCACACCAAUCACUAA.

### Luciferase assay

The DNA sequence of ITGB1 was amplified using the following primers: forward: 5′-GACGCCGCGCGGAAAAGATG-3′ and reverse: 5′-GCACCACCCACAATTTGGCCC-3′. The DNA sequence of the ITGA2 3′-UTR was amplified by PCR using the following primers: forward: 5′-ATAGGCCGGCATAGACGCGTACCAGCAGACCTACCTGCAG-3′ and reverse: 5′-AAAGATCCTTTATTAAGCTTACAGAGAAAATAAGGTGCAAAAAG-3′. The seed sequence was mutated using the following primers: forward: 5′-CTTCCCGTAACGGATTTTATTTATAAACTAGGTAAAATTTG-3′ and reverse: 5′-AAAGATCCTTTATTAAGCTTACAGAGAAAATAAGGTGCAAAAAG-3′. The amplified DNA sequences were embedded into the pmiR-REPORT™ Vector (OBIO, Shanghai, China) to generate ITGA2 and ITGB1 3′-UTR or mutated ITGA2 and ITGB1 3′-UTR luciferase vectors. To perform the dual-luciferase assay, 1.2 × 10^4^ 293 T cells in a 96-well plate were transfected with 100 nM miR-34c-3p or miR-NC (RiboBio). The cells were then cotransfected with 2 mg/mL of the vectors with the wild-type or mutant 3′-UTR of the ITGA2 or ITGB1 gene. After cells were transfected for 48 h, luciferase activity was examined using a Dual-Luciferase® Reporter Assay System (Promega, Madison, WI, USA). Herein, firefly luciferase activity was normalized to the comparable Renilla luciferase activity. All assays were performed in quadruplicate and were repeated three times.^40^

### Immunoprecipitation

Treated cells were collected and lysed in IP buffer (150 mM NaCl, 50 mM Tris HCl, 1% v/v Triton X-100 and 1 mM EDTA) containing phosphatase and protease inhibitors. Subsequently, the cell lysates (1 mg/mL) were incubated with antibodies against integrin α2 and integrin β1 overnight at 4 °C. After that, the cell lysates were incubated with Protein G Dynabeads at 4 °C for 3 h. After two washes with IP buffer, the proteins on the Dynabeads were eluted for 5 min by boiling at 95 °C in SDS sample buffer, and then they were separated by SDS-PAGE for subsequent Western blotting.^41^

### Immunohistochemistry

Paraffin-embedded tumor tissues were serially sectioned, deparaffinized in xylene and rehydrated with different concentrations of alcohol. Then, endogenous peroxidase was blocked by 3% v/v H_2_O_2_. After washing in distilled water, the indicated slides were incubated overnight with rabbit anti-integrin alpha 2 (ab133557, Abcam) diluted 1:250 or rabbit anti-integrin beta 1 (ab179471, Abcam) diluted 1:1000. DAB substrate was used to visualize immunoreactions. Slides were counterstained with hematoxylin and were mounted for observation under a microscope.^34^

### In vivo metastasis experiments

Five-week-old BALB/c nude mice were purchased from Tianqin Biotechnology Company (Changsha, China). All animals were bred and maintained in specific pathogen-free (SPF) conditions of the Center of Experimental Animal of Guangzhou Medical University. Experimental designs were approved by the Institutional Animal Care and Use Committee of Guangzhou Medical University. Mice were classified into three groups and were treated with different cells: untreated A549 cells and A549 cells incubated with 30 μg/mL and 60 μg/mL A549 exosomes for 24 h (10 mice per group, gender balanced). The above three A549 cells (2 × 10^6^ cells/mouse) were resuspended in PBS (200 μL/mouse) and injected intravenously (i.v.) into the mouse tail vein. Bodyweight was measured at least three times a week. After seven weeks, the mice were sacrificed. Their lungs were removed, immediately fixed in 4% (w/v) paraformaldehyde and embedded in paraffin for subsequent hematoxylin/eosin (HE) staining and immunohistochemical analysis.^42^ All experimental procedures followed these protocols, and rabbit anti-integrin alpha 2 (ab181548, Abcam) and rabbit anti-integrin beta 1 (ab179471, Abcam) were used in IHC.

### Statistical analysis

All experiments were performed in triplicate unless otherwise stated. All results are presented as the mean ± standard deviation (SD). SPSS 16.0 was used to analyze statistical differences in each assay. The significance was analyzed and tested by nonparametric tests and ANOVA, and then the relevance was analyzed by correlation analysis and chi-square tests. A *p-*value < 0.05 was regarded as statistically significant.

## Supplementary information

Figure S2

Supplementary Materials

Figure S1
