# Supplementary figures and images for: Exosomes with low miR-34c-3p expression promote invasion and migration of non-small cell lung cancer by upregulating integrin α2β1
Source: Signal Transduct Target Ther. 2020 Apr 22;5:39. doi: 10.1038/s41392-020-0133-y (PMC7174429; doi:10.1038/s41392-020-0133-y)

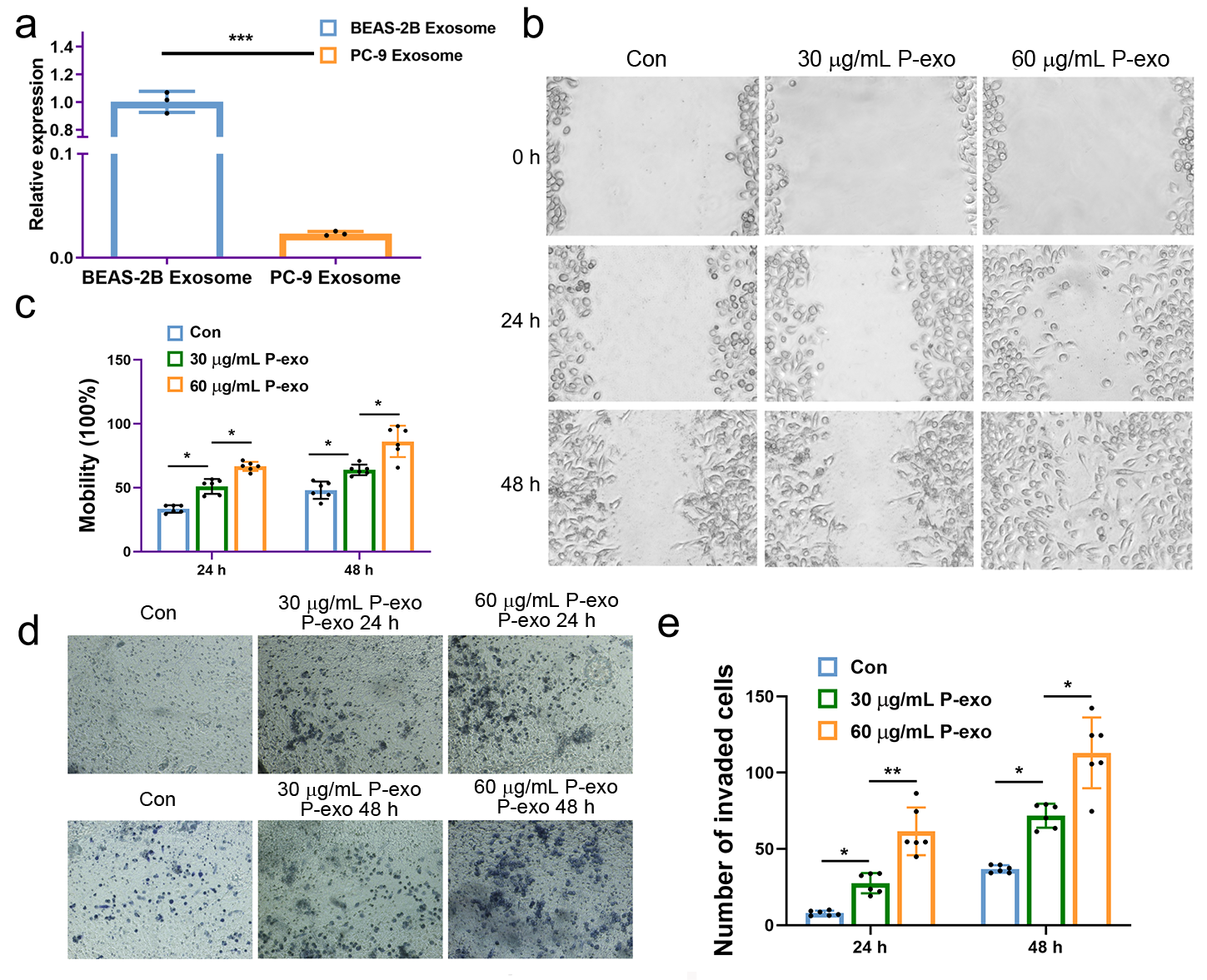

Supplement: Supplementary file 1 — Figure S2 [file 41392_2020_133_MOESM1_ESM.tif]

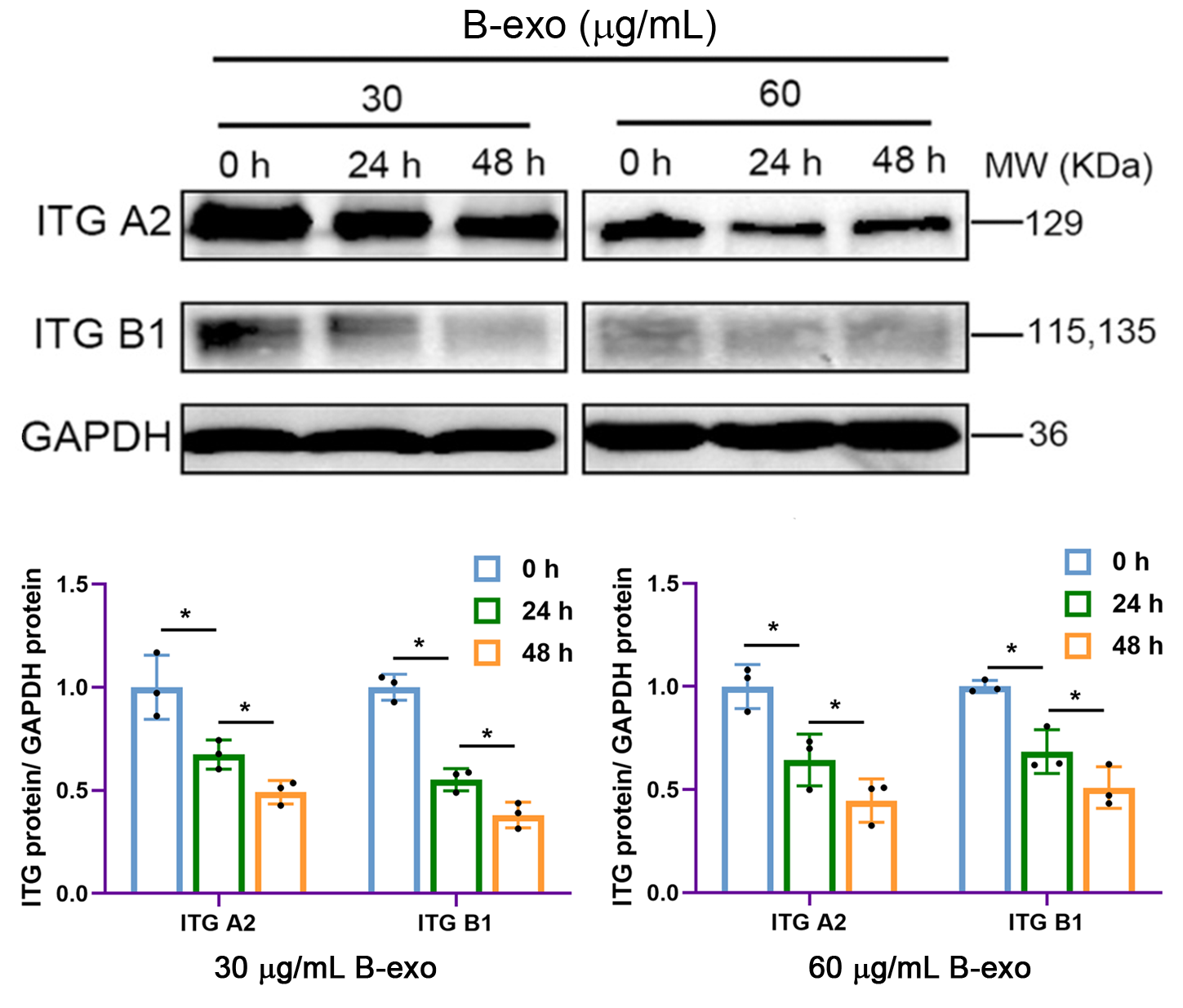

Supplement: Supplementary file 3 — Figure S1 [file 41392_2020_133_MOESM3_ESM.tif]
