# Supplementary material for: Exosomes with low miR-34c-3p expression promote invasion and migration of non-small cell lung cancer by upregulating integrin α2β1
Source: Signal Transduct Target Ther. 2020 Apr 22;5:39. doi: 10.1038/s41392-020-0133-y (PMC7174429; doi:10.1038/s41392-020-0133-y)
Supplement: Supplementary file 2 — Supplementary Materials [file 41392_2020_133_MOESM2_ESM.docx]

Supplementary Materials for

**Exosomes with low miR-34c-3p expression promote invasion and migration of non-small cell lung cancer by upregulating integrin α2β1**

Wenjing Huang^1, #^, Yanyan Yan^2, 3,^ ^#^, Yun Liu^1, #^, Minting Lin^1, #^, Jinxiang Ma^4^, Wei Zhang^5^, Jianwei Dai^6^, Jiajun Li^1^, Qiaoru Guo^1^, Hubiao Chen^3^, Bolat Makabel^7^, Hong Liu^2^, Chaoyue Su^1^, Hong Bi^8^, Jianye Zhang^1,9^ *

1. Guangdong Provincial Key Laboratory of Molecular Target & Clinical Pharmacology, School of Pharmaceutical Sciences and the Fifth Affiliated Hospital, Guangzhou Medical University, Guangzhou, Guangdong 511436, P.R.China.

2. Institute of Respiratory and Occupational Diseases, Collaborative Innovation Center for Cancer, Medical College, Shanxi Datong University, Datong 037009, P. R. China.

3. School of Chinese Medicine, Hong Kong Baptist University, Hong Kong, P.R.China.

4. College of Public Health, Guangzhou Medical University, Guangzhou, Guangdong 511436, P.R.China.

5. Cancer Center of Datong, the Second People’s Hospital of Datong, Shanxi 037005, P. R. China.

6. GZMU-GIBH School of life Sciences, Guangzhou Medical University, Guangzhou, Guangdong 511436, P.R.China.

7. Xinjiang Institute of Materia Medica, Urumqi 830004, P.R.China.

8. Department of Pathology, Shanxi Provincial People’s Hospital, Taiyuan 030012, P.R.China.

9. Key Laboratory of Tropical Translational Medicine of Ministry of Education, Hainan Medical University, Haikou 571199, P.R.China.

#: these authors contribute equally to this article.

*Corresponding author: Jianye Zhang, E-mail: jianyez@163.com

**This PDF file includes:**

Fig. S1 to S2

Fig. S1


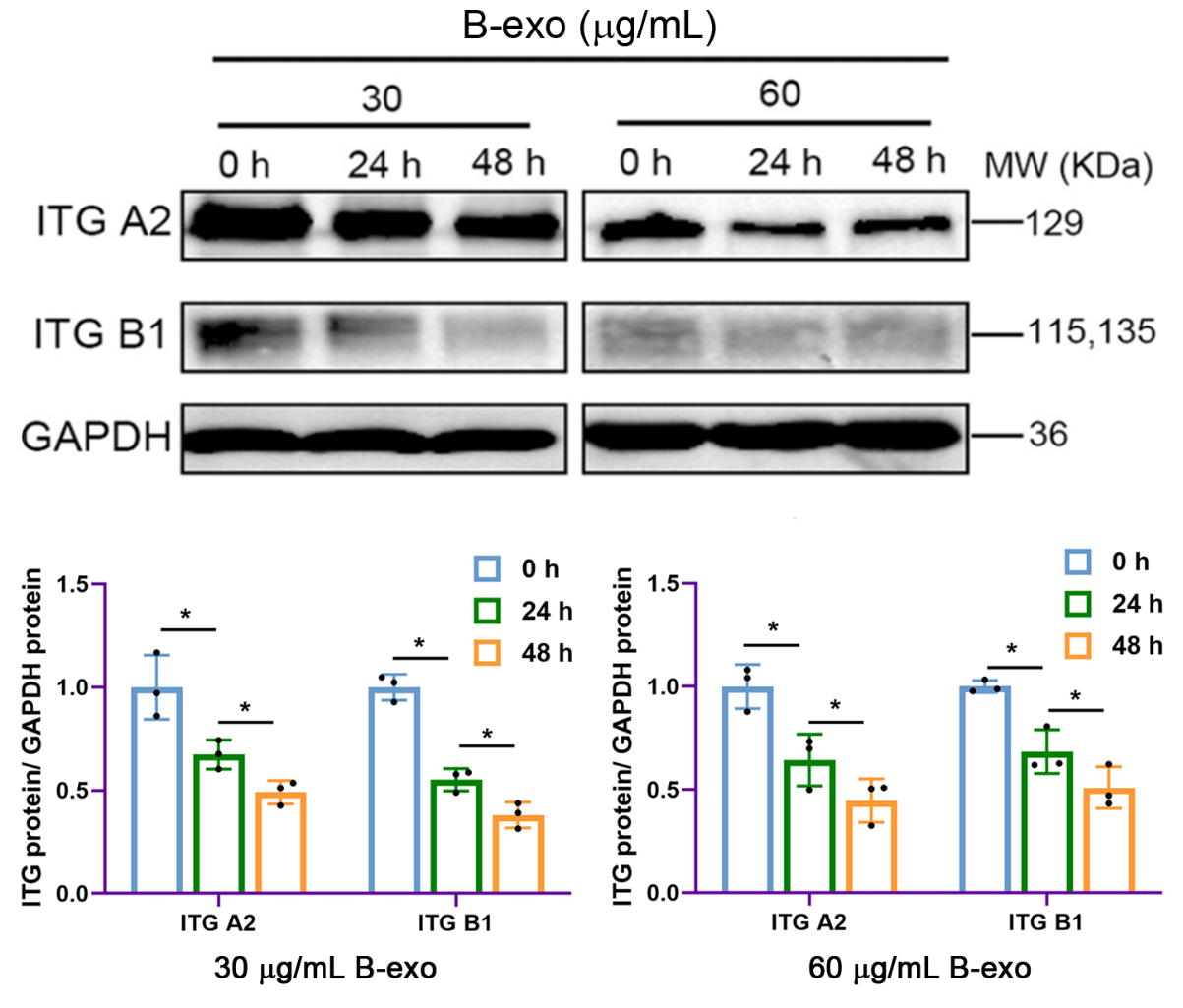


**Fig. S1 BEAS-2B derived-exosomes (B-exo) down-regulated the protein level of integrin α2β1 in A549 cells.**

Western blot assay for integrin α2β1 expression from different groups with BEAS-2B-exosomes (B-exo), and GAPDH as the normalization control. ITG A2= Integrin α2, ITG B1= Integrin β1.

Fig. S2


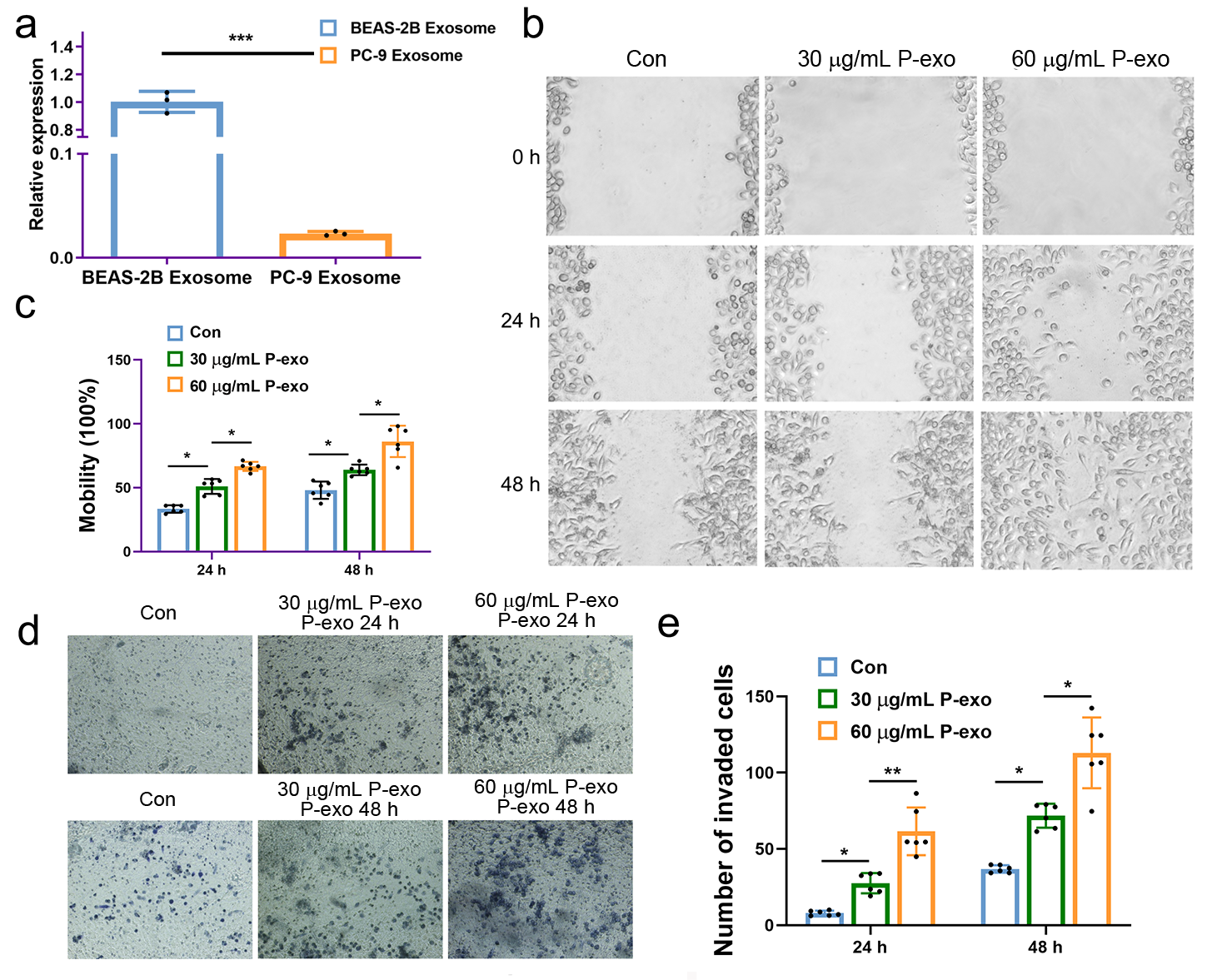


**Fig. S2 Increased invasion and migration of PC-9 cells following treatment with exosomes derived from NSCLC.**

**a** Compared with BEAS-2B derived-exosomes (B-exo), the level of miR-34c-3p was significantly lower in PC-9 derived-exosomes (P-exo). Experiments were performed in triplicates. **b** NSCLC PC-9 cell migration was induced by exosomes derived from PC-9 cells (P-exo) in a dose- and time-dependent manner. **c** Quantitative results of (b). ns = no significance, **P* < 0.05, ***p* < 0.01, n ≥ 3. **d** PC-9 cell invasion induced by PC-9 cells derived-exosomes (P-exo) in a dose- and time-dependent manner. **e** Quantitative results of (d). **p* < 0.05, ***p* < 0.01, n ≥ 3.
